# Assessing newborn body composition using principal components analysis: differences in the determinants of fat and skeletal size

**DOI:** 10.1186/1471-2431-6-24

**Published:** 2006-08-17

**Authors:** Beverley M Shields, Bridget A Knight, Roy J Powell, Andrew T Hattersley, David E Wright

**Affiliations:** 1 Peninsula Medical School, Barrack Road, Exeter, UK; 2 Heavitree Hospital, Royal Devon and Exeter NHS Foundation Trust, Exeter, UK; 3 Research and Development Support Unit, Royal Devon and Exeter NHS Foundation Trust Exeter, UK; 4 School of Mathematics and Statistics, University of Plymouth, Plymouth, UK

## Abstract

**Background:**

Birth weight is a composite of skeletal size and soft tissue. These components are likely to have different growth patterns. The aim of this paper is to investigate the association between established determinants of birth weight and these separate components.

**Methods:**

Weight, length, crown-rump, knee-heel, head circumference, arm circumference, and skinfold thicknesses were measured at birth in 699 healthy, term, UK babies recruited as part of the Exeter Family Study of Childhood Health. Corresponding measurements were taken on both parents. Principal components analysis with varimax rotation was used to reduce these measurements to two independent components each for mother, father and baby: one highly correlated with measures of fat, the other with skeletal size.

**Results:**

Gestational age was significantly related to skeletal size, in both boys and girls (r = 0.41 and 0.52), but not fat. Skeletal size at birth was also associated with parental skeletal size (maternal: r = 0.24 (boys), r = 0.39 (girls) ; paternal: r = 0.16 (boys), r = 0.25 (girls)), and maternal smoking (0.4 SD reduction in boys, 0.6 SD reduction in girls). Fat was associated with parity (first borns smaller by 0.45 SD in boys; 0.31 SD in girls), maternal glucose (r = 0.18 (boys); r = 0.27 (girls)) and maternal fat (r = 0.16 (boys); r = 0.36 (girls)).

**Conclusion:**

Principal components analysis with varimax rotation provides a useful method for reducing birth weight to two more meaningful components: skeletal size and fat. These components have different associations with known determinants of birth weight, suggesting fat and skeletal size may have different regulatory mechanisms, which would be important to consider when studying the associations of birth weight with later adult disease.

## Background

Birth weight, conventionally used as a measure of fetal growth, is a composite of many components including, bone, internal organs, muscle, fat and fluids. These may be determined by different regulatory mechanisms, both environmental and genetic. It has been suggested that fat-mass reflects the intra-uterine environment, whereas fat-free mass is more likely to be altered by genetic factors [1,2].

A major determinant of body composition at birth is gender. Males tend to be longer and heavier than females, but females have more subcutaneous fat [1,3-5]. Other factors known to influence body composition include parity and maternal BMI which have stronger associations with measures of fat at birth than measures of skeletal size [1,2], and maternal [2,6] and paternal [1,7,8] height which are more associated with skeletal size. The effects of smoking are less clear with some studies reporting a reduction only in skeletal measures as a result of maternal smoking [7,9-12], whereas others find a general decrease in all aspects of growth [13-15].

Identifying factors related to fetal growth has become an important part of the study of the developmental origins of adult disease. Relationships between low birth weight and later ill health such as diabetes, cardiovascular disease, stroke, and obesity, are well established [16-19]. Not only is birth weight seen to be important, but also how thin, or how short, the baby is [20-22]. Considering components of birth weight separately, and investigating their determinants, may give more insight into these associations.

There are various methods of obtaining measures of body composition in utero and at birth. Studies have used DEXA scans [7,23,24], ultransonography [25-27], and total body electrical conductivity [9,28]. These methods require expensive equipment, specialist training, and the more accurate methods are likely to be difficult to do in large samples. A simpler method for assessing body composition at birth is for midwives to take detailed anthropometry using standardized techniques, which can provide information on different aspects of growth. These measurements can be analysed individually [2,5,13,14,29]. However, often these measurements tend to be highly intercorrelated leading to problems of multicollinearity in regression analysis. Careful decisions, therefore, need to be made as to which measurements are the most appropriate and convey most information describing the aspect of growth of interest. A method able to combine them to provide more general estimates of size and body composition (as DEXA and ultrasonography do) would have considerable advantages. Mathematical equations to obtain estimates of body composition have been used previously, but these equations are not internally derived [1,30].

An alternative way of summarizing body size and composition using anthropometric measurements could be to use principal components analysis (PCA) [31], a method previously used to describe different aspects of birth size [10,23,32,33]. This statistical approach can be used to investigate the underlying structure of a dataset, by reducing the data to meaningful components. This method enables a large number of correlated variables to be summarized in terms of a relatively small number of uncorrelated principal components. The extent to which the principal components capture the variation in the original variables can be quantified in terms of the proportion of variance explained. The components can aid interpretation and represent meaningful constructs that parsimoniously describe the multivariate data.

We used principal components analysis to reduce multivariate measurements at birth to two more meaningful independent components representing different aspects of birth size: skeletal size and fat. We examined their relationships with established determinants of birth weight.

## Methods

### a) Subjects and methods

Families were recruited as part of the Exeter Family Study of Childhood Health [34], a large prospective study investigating genetic influences on fetal and early childhood growth. Parents were approached at the time of the ante-natal booking visit and invited to take part if they were Caucasian and living in central Exeter, as defined by postcode. Multiple pregnancies and women with diabetes were excluded. Both parents were required for the study. Informed written consent was obtained from the parents of the newborns and the study was approved by the North and East Devon Local Research Ethics Committee. In this paper we report the results of the first 800 families recruited between March 2000 and July 2003.

Babies were measured in detail within 24 hours of birth, in triplicate, by specially trained research midwives who underwent periodic inter-observer comparisons to ensure reliability and comparability. Birth weight was measured using a Soenhle scale and birth length was measured using the Harpenden Infantometer. Knee/heel length was measured using simple vernier calipers (to nearest 1 mm), and head and arm circumference with a short fiberglass tape (to nearest 1 mm). Skinfold thicknesses of the tricep and subscapular were taken using Holtain skinfold calipers (to nearest 0.2 mm).

Gestation was calculated based on last menstrual period (LMP) when periods were regular and where information was thought reliable (n = 369). Every woman had a dating scan around 12 weeks gestation (range 7–23 weeks) and gestation was determined by this method for women with irregular/unreliable menstrual cycles or if it differed from the LMP estimate by more than 10 days (n = 330).

Detailed anthropometric measurements were taken on both parents at 28 weeks gestation by the research midwives. Weight was measured using Tanita electric scales (to nearest 0.1 kg). A Harpenden pocket stadiometer was used for height and sitting-height measurements, and simple vernier calipers (to nearest 0.1 cm) were used for knee-heel length. Head and arm circumference were measured using a short, non-stretchable fiberglass tape (to nearest 0.1 cm), and a strong fiberglass tape (range up to 150 cm) was used for waist and hip measurements in the father. Skinfold thicknesses of the bicep, triceps and subscapula, plus the suprailiac in the father only were taken on the non-dominant side of the body (to nearest 0.2 mm using Holtain skinfold calipers). Coefficient of variation (CV) between midwives was less than 1% for weight and linear measures, and less than 5% for measures of skinfold thickness.

Fasting plasma glucose concentration was measured in the mother, also at 28 weeks gestation, using standard laboratory methods carried out by the pathology laboratories at the Royal Devon & Exeter Hospital, Exeter, UK.

### b) Statistics

Distributions of individual variables were assessed for normality and in the case of skinfold thicknesses and weight, log transformations were applied. For these variables, geometric means are presented.

Principal components analysis was carried out on the anthropometric measurements from both parents and their babies at birth. The components analysis was based on correlation matrices rather than covariance matrices as the variables were on different scales of measurement. Varimax rotation was applied to transform the original principal components produced, to ease interpretation. This method searches for a linear combination of the original measurements aiming to maximize the variance of the component loadings, leading to high correlations with some of the original variables, and low correlations with others. As the associations between the rotated components and the original variables are on scales of 0 to 1, it becomes easier to define them.

Interpretable principal components scores were produced summarizing the multivariate data in terms of standardized variables with zero mean and standard deviation of one. Recognising the differences in anthropometry between the sexes, boys and girls were analysed separately. Components were also produced for both parents.

T tests were used to examine the effect of parity (primiparous or multiparous) and smoking. Pearson correlation coefficients were used to assess relationships of birth components with gestation, maternal fasting glucose, and parental size components. Multiple linear regression analysis was applied to determine independent predictors of the birth components.

## Results

### a) Study cohort (Fig 1)

**Figure 1:**
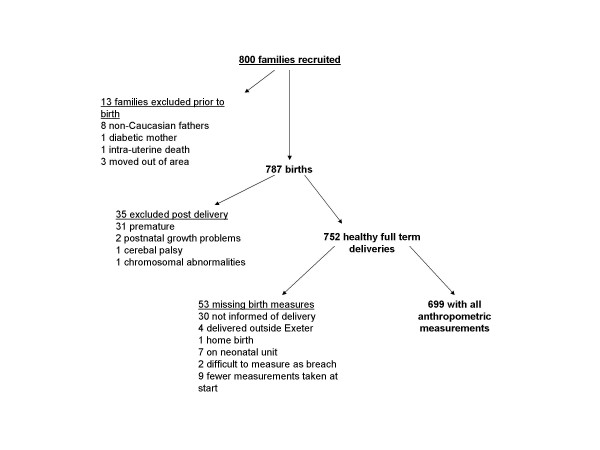
Flow diagram showing families selected for analysis.

Disposition of the 800 families initially recruited into the EFSOCH study is presented in Figure 1. The analysis presented in this paper focuses on the 752 healthy full term babies. Full anthropometric data were unavailable for 53 (7%) of these babies. There was no evidence of any differences in birth weight and gestation between those with measures available and those without (birth weight: 3521 g v 3511 g, p = 0.893, respectively; gestation: 40.2 wks v 39.9 wks, p = 0.101, respectively).

The babies' birth anthropometry is summarized in Table 1. Boys were heavier and longer than girls, but girls had greater skinfold thicknesses and ponderal index.

**Table 1:** Characteristics of EFSOCH babies at birth.

	Boys (mean +/- SD)	Girls (mean +/- SD)	p
n	363	336	
Gestation (weeks)	40.1 +/- 1.14	40.2 +/- 1.27	0.1
Weight (g)	3559 +/- 461	3479 +/- 482	0.02
Length (cm)	50.5 +/- 2.0	49.7 +/- 2.1	<0.001
Crown rump (cm)	34.1 +/- 1.6	33.7 +/- 1.7	0.001
Knee heel (cm)	12.6 +/- 0.6	12.4 +/- 0.6	0.001
Head circ (cm)	35.5 +/- 1.2	34.9 +/- 1.3	<0.001
Arm circ (cm)	11.1 +/- 0.9	11.1 +/- 0.9	0.9
Tricep (mm)*	4.6 (3.7–5.8)	4.8 (3.8–6.1)	0.005
Subscapula (mm)*	4.7 (3.7–6.0)	4.9 (3.8–6.2)	0.005
Ponderal index (kg/m^3^)	27.5 +/- 2.6	28.1 +/- 2.6	

*Log transformed data – geometric mean and SD range reported

Table 2 shows the characteristics of the parents. Mothers had a mean age of 30 years and a mean reported pre-pregnant BMI of 24 kg/m^2^. 88 (12.6%) of mothers smoked. 299 (42.8%) were primiparous. Fathers had a mean age of 33 years with a mean BMI of 27 kg/m^2^.

**Table 2:** Characteristics of parents in EFSOCH.

	Mothers (mean +/- SD)	Fathers (mean +/- SD)
n	699	699
Age (years)	30 +/- 5.2	33 +/- 6.0
Weight (kg)*	74.7 (63.5–88.0)	83.4 (70.9–98.1)
Height (cm)	164.9 +/- 6.3	177.8 +/- 6.7
BMI (kg/m^2^)	27.9 +/- 4.7	26.7 +/- 3.9
Knee heel (cm)	49.5 +/- 2.5	53.9 +/- 2.6
Sitting Height (cm)	85.3 +/- 3.4	90.5 +/- 3.5
Head Circumference (cm)	55.8 +/- 1.5	58.3 +/- 1.6
Arm Circumference (cm)	29.0 +/- 3.5	31.7 +/- 3.1
Bicep skinfold thickness (mm)*	11.1 (7.4–16.7)	6.5 (4.1–10.1)
Tricep skinfold thickness (mm)*	20.1 (14.4–28.2)	10.5 (6.7–16.5)
Subscapular skinfold thickness (mm)*	20.1 (13.4–30.1)	17.0 (11.0–26.3)
Suprailiac skinfold thickness (mm)	N/A	26.6 +/- 10.3
Waist circumference (cm)	N/A	92.2 +/- 11.1
Hip circumference (cm)	N/A	104.6 +/- 7.0

* Log transformed data – geometric mean and SD range reported

### b) Principal components analysis

All birth measures were entered into a principal components analysis. In each case the birth measurements were reduced to two components.

The principal components analyses, showing the unrotated components for boys and girls, are given in Table 3. In both boys and girls, the first unrotated principal component (B1 and G1), which individually explained the largest proportion of variance possible, had the strongest correlation with birth weight and was highly correlated with all measures of birth size. This can be interpreted as a general measure of birth size and it explained 61.7% and 65.4% of the variance for boys and girls, respectively. The second unrotated principal component (B2 and G2) was also similar in boys and girls and showed negative correlations with measures of skeletal size and positive correlations with measures of fat, and we interpreted the contrast in this component as representing body composition, explaining 15.7% and 13.2% of the variance, respectively. In both boys and girls, the two components together explained almost 80% of the variation in the original 8 variables.

**Table 3:** Unrotated components matrix for principal components analysis of boys' and girls' birth anthropometry.

	Boys		Girls	
Component:	B1	B2	G1	G2

Birth weight	0.951	-0.012	0.949	-0.027
Length	0.803	-0.426	0.834	-0.398
Crown Rump	0.842	-0.262	0.834	-0.305
Knee Heel	0.702	-0.456	0.769	-0.322
Head Circumference	0.743	-0.166	0.780	-0.129
Arm Circumference	0.843	0.254	0.867	0.181
Tricep skinfold	0.696	0.564	0.723	0.533
Subscapular skinfold	0.658	0.625	0.684	0.608

Variance (%)	61.7	15.7	65.4	13.2

Cumulative Variance (%)	77.4		78.7	

Values shown are component loadings (correlation coefficients of the relationship between the components produced (B1, B2, G1 and G2) and the individual measurements). B1 represents the first unrotated component produced for the boys, B2 represents the second unrotated component produced for the boys. G1 represents the first unrotated component produced for the girls, G2 represents the second unrotated component produced for the girls. Variance is the percentage variance explained by each component. Cumulative Variance indicates the variance explained by both components together.

These components failed to capture clinically distinct constructs, therefore, varimax rotation was performed (Table 4). This rotated analysis led to the identification of 2 components for both boys and girls, explaining the same cumulative variance as the components from the first analysis. Component 1 (B1R and G1R) has high correlations with the skeletal measurements of length, crown-rump and knee heel (r = 0.82-0.90), and low correlations with tricep and subscapular skinfold thicknesses (fat measures) (r = 0.13-0.20). Therefore, we interpreted this component as a "skeletal size" component. This is in contrast to the second rotated component produced (B2R and G2R) which has high correlations with the skinfold thicknesses (r = 0.85-0.90) and low correlations with measures of skeletal size (r = 0.18-0.26), which we interpreted as a "fat" component. Birth weight and arm circumference have high correlations (r > 0.5) with both components in the boys and girls analyses and this fits in with our interpretation of the components as they are measurements of both skeletal size and fat. More of the variation in the skeletal size component could be explained by the variables entered into the principal components analysis compared with the fat component (44% and 46.7% v 33.4% and 31.9%).

**Table 4:** Varimax rotated components matrix for principal components analysis of boys' and girls' birth anthropometry.

	Boys		Girls	
Component:	B1R	B2R	G1R	G2R

Birth weight	0.753	0.581	0.776	0.546
Length	0.895	0.164	0.906	0.181
Crown Rump	0.823	0.317	0.850	0.255
Knee Heel	0.834	0.078	0.809	0.202
Head Circumference	0.686	0.331	0.701	0.364
Arm Circumference	0.504	0.722	0.586	0.664
Tricep skinfold	0.196	0.873	0.260	0.860
Subscapular skinfold	0.129	0.898	0.184	0.897

%Variance	44.0	33.4	46.7	31.9

Cumulative Variance (%)	77.4		78.7	

Values shown are component loadings (correlation coefficients of the relationship between the components produced (B1R, B2R, G1R and G2R) and the individual measurements). B1R represents the first rotated component produced for the boys, B2R represents the second rotated component produced for the boys. G1R represents the first rotated component produced for the girls, G2R represents the second rotated component produced for the girls. Variance is the percentage variance explained by each component. Cumulative Variance indicates the variance explained by both components together.

**Figure 2:**
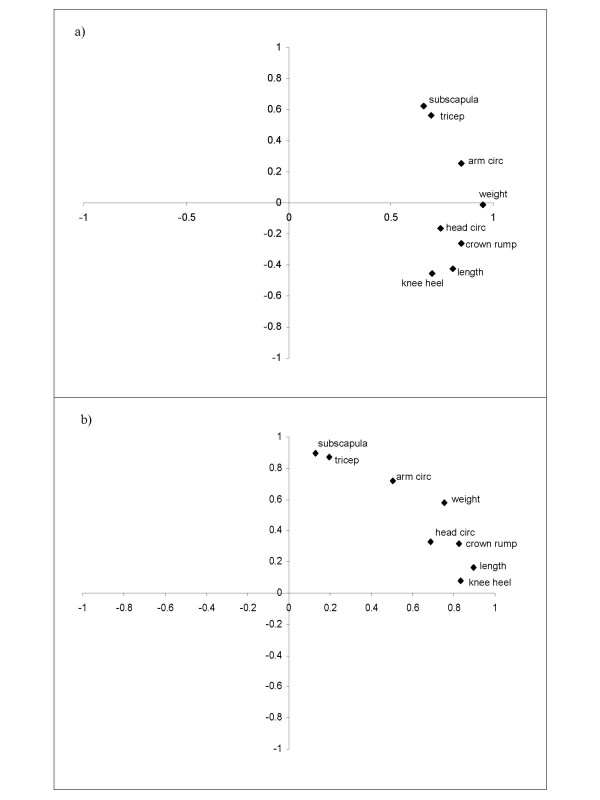
Scatterplots of the component loadings for the a) unrotated and b) rotated principal components analysis of the child's birth measurements.

To further explain the method of varimax rotation, figure 2 shows plots of the unrotated and rotated component loadings for the child's birth measurements. It can clearly be seen that the relative location of the points on the rotated solution remains the same, but the axes have been rotated so that the variance of the coefficients is maximised. The "fat" measures are closest to the y axis, whereas the "skeletal" measures are closest to the x axis.

Principal Components Analysis was also used to reduce the anthropometric measurements in the parents (Rotated components matrix shown in Table 5). Components similar to the babies' were produced, but this time the fat component in both mothers and fathers (M2 and F2) explained more of the variance in the measurements than the skeletal size component (M1 and F1) (43.4 and 48.1% v 30.8 and 25.9%). The parents' components were more distinct with the lowest correlation coefficients being negative and close to zero, and the highest being greater than 0.9.

**Table 5:** Rotated components matrix for principal components analysis of mothers' and fathers' anthropometry.

	Mothers		Fathers	
Component:	M1	M2	F1	F2

Weight	0.413	0.844	0.514	0.810
Height	0.936	-0.100	0.953	-0.018
Knee-Heel	0.834	-0.012	0.873	0.065
Sitting height	0.816	0.032	0.807	0.133
Head circumference	0.595	0.190	0.489	0.334
Arm circumference	0.098	0.928	0.241	0.786
Bicep skinfold	-0.020	0.878	0.044	0.844
Tricep skinfold	-0.060	0.871	0.021	0.768
Subscapular skinfold	-0.040	0.867	0.002	0.912
Suprailiac skinfold	-	-	0.034	0.864
Waist circumference	-	-	0.228	0.895
Hip circumference	-	-	0.405	0.823

% Variance	30.8	43.4	25.9	48.1

Cumulative %Variance	74.2		73.9	

Values shown are component loadings (correlation coefficients of the relationship between the components produced (M1, M2, F1 and F2) and the individual measurements). M1 represents the first rotated component produced for the mothers, M2 represents the second rotated component produced for the mothers. F1 represents the first rotated component produced for the fathers, F2 represents the second rotated component produced for the fathers. Variance is the percentage variance explained by each component. Cumulative Variance indicates the variance explained by both components together.

### c) Associations with skeletal size and fat at birth

The rotated components at birth were examined for associations with characteristics known to be associated with birth weight.

#### i) Categorical variables: birth order and smoking

**Figure 3:**
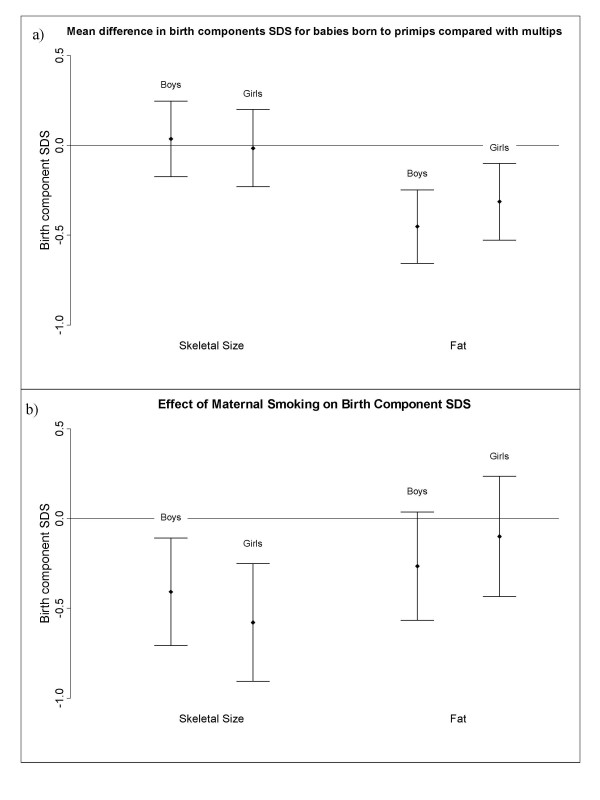
Figure 3a shows the mean difference and 95% confidence intervals for birth skeletal size and birth fat components, expressed as standard deviations, for babies born to primips compared with babies born to multips, boys and girls shown separately. Fig. 3b shows the mean difference and 95% confidence intervals for birth skeletal size and birth fat components, expressed as standard deviations, for babies born to smoking mothers, compared with non-smoking mothers, boys and girls shown separately. Birth component score is an internally derived standard deviation score from the principal components analysis.

Parity was associated with the fat component but not the skeletal size component in both boys and girls (Fig. 3a), with babies born to primips having less fat than babies born to multips (first born babies: 0.45 SD (95%CI: 0.24–0.66) lower in boys, p < 0.001; 0.31 SD (95%CI: 0.10–0.53) lower in girls, p = 0.004). Maternal smoking during pregnancy resulted in a reduction in the skeletal size component (0.41 SD (95% CI: 0.10–0.72) decrease for boys (p = 0.008) and 0.58 SD (95%CI: 0.24–0.91) decrease for girls (p < 0.001)) but there was no significant effect on fat for both boys and girls (Fig. 3b).

#### ii) Continuous variables: gestation, maternal glucose and parental size

**Figure 4:**
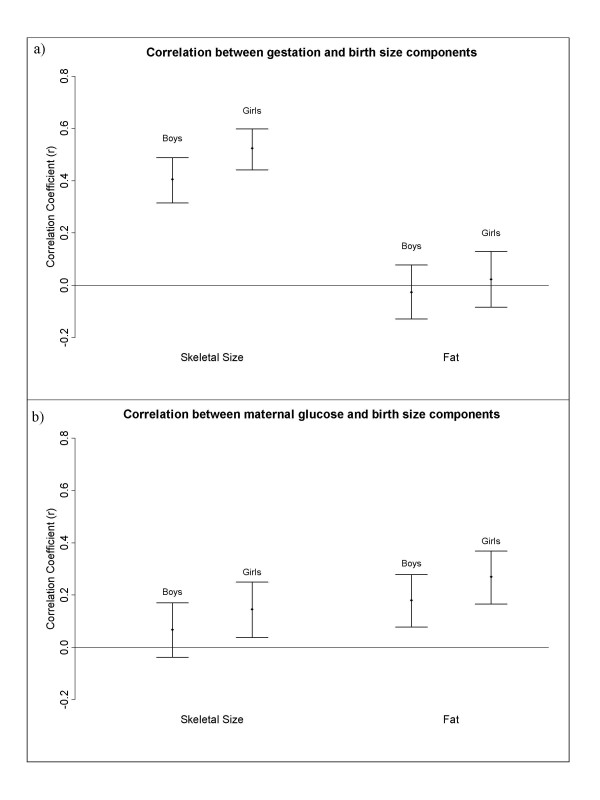
Figure 4a shows the correlation coefficient and 95% confidence intervals for the relationship between gestation and birth size components, boys and girls shown separately. Figure 4b shows the correlation coefficient and 95% confidence intervals for the relationship between maternal 28 week fasting glucose and birth size components, boys and girls shown separately.

Gestation was associated with only the skeletal size component in both boys and girls (r = 0.41 and r = 0.52, respectively), but not the fat component (r = -0.03 and 0.02) (Fig. 4a). Maternal glucose was significantly correlated with both components in girls (r = 0.15 for fat and r = 0.27 for skeletal size), but only fat (r = 0.18) in boys (Fig 4b).

Table 6 shows the relationship between the birth size components and parents' size components. The skeletal size component in both parents was associated with the birth skeletal size component in both boys and girls (r = 0.16-0.39), although the association between paternal and boys' skeletal size did not quite reach significance when Bonferroni adjustments were made for multiple testing (p = 0.06). Maternal fat component was correlated with fat at birth (r = 0.16 and 0.36), but not skeletal size (r = 0.07 and 0.05) in both boys and girls. Paternal fat was not correlated with fat or skeletal size in either sex (r = 0.05-0.10).

**Table 6:** Determinants of birth skeletal size and fat in boys and girls:

	Boys skeletal size	Girls skeletal size	Boys fat	Girls fat
Maternal skeletal component	0.235*** (0.055)	0.390*** (0.152)	-0.150 (0.023)	0.050 (0.003)
Maternal fat component	0.071 (0.005)	0.053 (0.003)	0.163* (0.027)	0.357*** (0.127)
Paternal skeletal component	0.164 (0.027)	0.250*** (0.063)	-0.048 (0.002)	-0.033 (0.001)
Paternal fat component	0.095 (0.009)	0.045 (0.002)	0.095 (0.009)	0.056 (0.003)

Correlation coefficients (r) of relationships between birth components and parents' components. R^2 ^is given in brackets. ***p < 0.001, *p < 0.05.(based on Bonferroni adjusted p values).

#### iii) Multiple linear regression analysis

Multiple linear regression was used to identify independent predictors of skeletal size and fat components in the boys and girls (Table 7). Four analyses were performed, with skeletal size and fat components as the dependent variables for boys and girls separately.

**Table 7:** Multiple regression analysis of predictors of boys and girls skeletal size and fat components. Only factors with p < 0.1 are shown.

Covariate	B	SE	t	p	R^2^
Boys skeletal size					0.278
Gestation	0.372	0.044	8.5	<0.001	
Maternal Skeletal Size	0.215	0.053	4.1	<0.001	
Paternal Skeletal Size	0.148	0.053	2.8	0.005	
Maternal Smoking	-0.406	0.151	-2.7	0.008	
Parity (primip)	-0.211	0.102	-2.1	0.04	

Girls' Skeletal Size					0.428
Gestation	0.354	0.036	9.7	<0.001	
Maternal Skeletal Size	0.266	0.044	6.0	<0.001	
Paternal Skeletal Size	0.169	0.045	3.7	<0.001	
Maternal Glucose	0.414	0.137	3.0	0.003	
Maternal Smoking	-0.301	0.139	-2.2	0.03	
Parity (Primip)	-0.170	0.090	-1.9	0.06	

Boys Fat					0.158
Parity (Primip)	-0.461	0.110	-4.2	<0.001	
Maternal Smoking	-0.646	0.164	-3.9	<0.001	
Maternal Skeletal Size	-0.141	0.057	-2.4	0.02	
Maternal Glucose	0.322	0.158	2.0	0.04	
Maternal Fat	0.107	0.059	1.8	0.07	

Girls' Fat					0.189
Maternal Fat	0.302	0.057	5.3	<0.001	
Maternal Glucose	0.476	0.171	2.78	0.006	
Parity	-0.283	0.113	-2.5	0.01	

Data sorted according to t value to show strongest associations first. B is the regression coefficient. SE is the standard deviation for the regression coefficient.

The strongest independent associations found with skeletal size components in both sexes were gestation, and maternal and paternal skeletal size. Other significant independent predictors of the skeletal size component were maternal glucose in the girls only, parity in the boys (which did not quite reach significance in the girls) and maternal smoking which showed a negative association in both.

Parity and maternal glucose showed independent associations with the fat component in both boys and girls. The strongest independent predictor of the girls' fat component was maternal fat, which did not reach significance in the boys' analysis. Maternal smoking and maternal skeletal size both showed negative independent associations with the boys' fat component, but there was no association with the girls'.

Data were assessed for model fit, and apart from one outlying observation in the skeletal data for boys and for girls, there was no evidence of any departure from the model assumptions. The removal of these outliers did not substantially alter the conclusions.

## Discussion

Principal components analysis produced two clear components allowing recognition of discrete aspects of birth size when using the rotated analysis. The rotated components, which can be considered to represent "skeletal size" and "fat", had different associations with factors known to alter birth weight.

There are various methods of obtaining measures of fat and skeletal size in utero and at birth. Direct measures of body composition can be obtained using DEXA scans [7], ultransonography [25-27], and total body electrical conductivity [9,28]. Although DEXA and ultrasonography would be the gold standard for measuring body composition, these methods are likely to be expensive and impractical in a large epidemiological study. Anthropometric measurements provide an alternative method where measures such as head circumference, length, and limb length can represent skeletal size, and measures of skinfold thicknesses can represent fat. Mathematical equations for combining these measurements to obtain estimates of body composition have previously been defined and can be applied retrospectively to the data [35,36]. Principal components analysis is another way of summarizing body size and composition using these anthropometric measurements and enables easier analysis of the data by reducing the problem of multicollinearity. It is a well established statistical method that can easily be implemented and allows the components to be internally derived from the data.

It has been suggested that genetic factors are more likely to alter skeletal size whereas the intrauterine environment has more of an effect on fat mass[1]. We were able to gain some insight into potential genetic determinants by also producing components for the mother and father, which had not been carried out in other studies looking at PCA on birth size. Maternal and paternal skeletal size components were found to have significant associations with the skeletal size components in boys and girls (Tables 6 and 7), in keeping with the notion that genetic factors are more likely to affect skeletal growth. This finding is consistent with associations found between parental height and birth length seen in other studies [2,6-8,10].

Factors associated with the fat component were largely those related to the intrauterine environment (Table 7) with parity and maternal glucose associated with fat in both boys and girls, and the strongest predictor of fat in the girls being maternal fat component (Table 7). Similar relationships of parity with fat measures have recently been reported [1,2] and it has been suggested this is due to the mothers' vascular system becoming better adapted to the transfer of energy to the fetus after the first pregnancy [2]. Our findings are also in agreement with other studies that have found maternal glucose [27,28] and maternal BMI [2,37] to be good predictors of measures of fat and soft tissue in babies at birth.

Maternal smoking appeared to alter only the skeletal size component (Fig 3b), suggesting the intra-uterine environment does play a role in determining skeletal size. This is consistent with previous research [7,9,10], particularly the studies by Lindley et al[11,12] who found reduced birth length, and head circumference, but increased ponderal index in babies born to smoking mothers, suggesting fat deposition is maintained despite deficits in other aspects of fetal growth. Other studies have found maternal smoking to be associated with a reduction in all measures of size, including fat and skinfold thicknesses [11,13,14], although the relationship with fat reported by Zaren et al[13] was weak, and in the study by Cliver et al[14], skinfolds were only affected in babies born to heavy smokers. In multiple regression analysis (Table 7) smoking did show an independent association with the fat component in the boys, but not the girls, which may account for some of the differences in the literature. This sexual dimorphism has been seen before [15] and it was suggested that it may be due to hormonal differences.

These findings are all consistent with previous research. The disparity between the associations of gestation and the components of birth size, however, has not been described previously. In our data the strongest independent predictor of the skeletal size component in both boys and girls was gestation, but it showed no association with the fat component. It is important to note these results are only based on term babies, so gestation in this study refers to the limited period of 37–42 weeks. Our data suggest that after 37 weeks gestation the increase in birth weight is as a result of further skeletal growth, rather than fat deposition (Fig 4a and Table 7). In contrast, other studies have found increases in skinfold thickness [3,5,38] and fat mass [25] with gestational age at term. In our data, the subscapular skinfold thickness had a weak but significant correlation with gestation (r = 0.096, p = 0.011), but the tricep skinfold thickness did not (r = 0.067, p = 0.076). These differences may be partly due to what the components represent. A recent study by Guihard-Costa et al. [29] found that although skinfold thicknesses increased with gestation, their ratio with body weight between 33 and 42 weeks gestation significantly decreased, and this may reflect what is seen with our data. This finding warrants further investigation as our cross-sectional data are not conclusive.

Other studies have used principal components analysis to produce components describing birth size [10,32,33], although they differ slightly from the components produced from our data. Denham et al[32] used principal components analysis to describe differences in neonatal size between black and white babies of low socioeconomic status. The components they obtained were similar to the unrotated components produced in our data (Table 3), representing general body size, and contrasts between skeletal and fat measures (referred to as "body composition"). Hindmarsh et al[10] examined size and shape at birth and reported 4 components in total, although the third and fourth components together explained only an additional 15% of the variation in birth measures. The first two components again were similar to our unrotated components. A key difference in our analysis, was the use of varimax rotation on the components (Table 4). The advantage of this method over the unrotated analysis, is that we obtained a clear distinction between measures of skeletal size and fat which are more meaningful in terms of explaining body size. Components representing skeletal size and fat were produced in the study by Evans et al[33] investigating the effect of frequent prenatal ultrasound examinations on birthweight. However, their components were obtained by a slightly different method: two separate analyses were carried out, one entering in only skeletal measures, and one entering in only fat measures, rather than all measures being entered into one analysis. Similarly, Koo et al[23] derived two components representing fat: one from PCA on neonatal body circumferences, and one from PCA on skinfold thickness measurements. The advantage of our method is that no clinical assumptions need to be made about the data prior to analysis. The components are derived purely from the data.

It is clear that this analysis will need to be repeated in another population, particularly as the difference in the effect of gestation on skeletal size and fat is not widely reported in the literature. Further research into how representative the components are of skeletal size and fat compared with more complex measures of body composition would also be of interest. However, other associations found with the components largely confirm what has been found in other studies looking at determinants of body composition, suggesting principal components analysis is a valid method for analysing birth size.

## Conclusion

In conclusion, we have provided further evidence demonstrating principal components analysis is a useful method for analysing anthropometric data in babies at birth. The addition of varimax rotation enabled more distinct, clinically relevant, components to be produced than those seen in previous studies. We have clearly shown differences in the determinants of skeletal size and fat components at birth, which support findings from previous research, as well as highlighting possible new areas for further study. This approach may be a useful tool in future investigations into the developmental origins of adult disease.

## Competing interests

The author(s) declare that they have no competing interests.

## Authors' contributions

BS carried out the statistical analysis and drafted the manuscript. BK recruited subjects, carried out measurements and also assisted in the writing of the manuscript. RP advised on statistics and helped to draft the manuscript. AH conceived of the Exeter Family Study of Childhood Health and helped to plan and write the manuscript. DW conceived of the idea of using Principal Components Analysis to analyse the birth measurements, provided statistical support in the analyzing of the data, and helped to draft the manuscript. All authors read and approved the final manuscript.

## Pre-publication history

The pre-publication history for this paper can be accessed here:


